# Moving GPU‐OpenCL‐based Monte Carlo dose calculation toward clinical use: Automatic beam commissioning and source sampling for treatment plan dose calculation

**DOI:** 10.1002/acm2.12049

**Published:** 2017-02-16

**Authors:** Zhen Tian, Yongbao Li, Nima Hassan‐Rezaeian, Steve B. Jiang, Xun Jia

**Affiliations:** ^1^ Department of Radiation Oncology University of Texas Southwestern Medical Center Dallas TX 75390 USA; ^2^ School of Astronautics Beihang University Beijing 100191 China

**Keywords:** automatic beam commissioning, GPU‐based Monte Carlo dose calculation, source sampling, VMAT dose calculation

## Abstract

We have previously developed a GPU‐based Monte Carlo (MC) dose engine on the OpenCL platform, named goMC, with a built‐in analytical linear accelerator (linac) beam model. In this paper, we report our recent improvement on goMC to move it toward clinical use. First, we have adapted a previously developed automatic beam commissioning approach to our beam model. The commissioning was conducted through an optimization process, minimizing the discrepancies between calculated dose and measurement. We successfully commissioned six beam models built for Varian TrueBeam linac photon beams, including four beams of different energies (6 MV, 10 MV, 15 MV, and 18 MV) and two flattening‐filter‐free (FFF) beams of 6 MV and 10 MV. Second, to facilitate the use of goMC for treatment plan dose calculations, we have developed an efficient source particle sampling strategy. It uses the pre‐generated fluence maps (FMs) to bias the sampling of the control point for source particles already sampled from our beam model. It could effectively reduce the number of source particles required to reach a statistical uncertainty level in the calculated dose, as compared to the conventional FM weighting method. For a head‐and‐neck patient treated with volumetric modulated arc therapy (VMAT), a reduction factor of ~2.8 was achieved, accelerating dose calculation from 150.9 s to 51.5 s. The overall accuracy of goMC was investigated on a VMAT prostate patient case treated with 10 MV FFF beam. 3D gamma index test was conducted to evaluate the discrepancy between our calculated dose and the dose calculated in Varian Eclipse treatment planning system. The passing rate was 99.82% for 2%/2 mm criterion and 95.71% for 1%/1 mm criterion. Our studies have demonstrated the effectiveness and feasibility of our auto‐commissioning approach and new source sampling strategy for fast and accurate MC dose calculations for treatment plans.

## 1 Introduction

Although Monte Carlo (MC) simulation is the most accurate dose calculation method in radiotherapy,^1^ its long computational time has prevented clinical application. Recent research efforts have focused on developing high performance MC simulation packages on graphics processing unit (GPU) platforms.^2^, ^3^, ^4^, ^5^, ^6^, ^7^, ^8^ While the accuracy and efficiency of fast GPU‐based particle transport simulations in patients have been demonstrated, a number of barriers still need to be overcome before using GPU‐based MC dose calculations in the clinic.

An accurate linear accelerator (linac) beam model is critical for the overall accuracy of MC dose calculations for external radiotherapy.^1^, ^9^, ^10^, ^11^ A phase‐space file obtained from the MC simulation of a linac head is the most accurate beam model, and hence is used in many CPU‐based MC packages. However, regarding the computational efficiency, it is not preferable to directly use a phase‐space file in GPU‐based MC simulation. One reason is the overhead of loading a large number of particles and transferring them from CPU to GPU. Although the data loading and transferring time (usually in seconds) is not significant for traditional CPU‐based MC calculations, it becomes a considerable issue in GPU‐based simulation when compared with the short simulation time on GPU (less than 1 minute for a typical patient case).^12^ In addition, direct use of a phase‐space file is not suitable for GPU's single‐instruction‐multiple‐data programming scheme. That is because particles are stored in the phase‐space files in a random order in terms of particle type (photon or electron), energy, and spatial location. When simulating a group of particles of different types and energies simultaneously on GPU platforms, the threads inside a warp will have very different execution paths, leading to the so‐called thread divergence issue and impairing the overall program efficiency.^8^ Fine‐tuning the beam model to properly represent an actual clinical beam is an essential step. However, a phase‐space file cannot be easily commissioned to represent a specific clinical beam. Conventional solution is to tune the parameters in linac head MC simulations in a trial‐and‐error fashion. Besides being cumbersome and time‐consuming,^13^, ^14^, ^15^, ^16^ this commissioning process requires MC expertise, impeding its application in the clinic. An accurate beam model with an easy commissioning approach is hence required to facilitate the clinical application of MC dose calculations.

We have recently developed a GPU‐OpenCL‐based MC dose engine named goMC for radiotherapy, a cross‐platform MC dose engine that may be executed on different computing devices, such as CPU, GPU from different vendors, and heterogeneous systems.^7^ An analytical linac beam model derived from a phase‐space file has been added to goMC for external photon therapy, along with a GPU‐friendly scheme to sample source particles from the model.^17^ In this paper, we report our effort in moving goMC toward clinical use. Regarding beam commissioning, we have previously developed an optimization‐based automatic commissioning approach for a phase‐space‐file‐based beam model.^18^ We have shown that using measurement dose in water, this approach can finely tune the energy spectrum and fluence distribution of the beam model to accurately represent the targeted beam. The accuracy of the commissioned beam model was validated in water as well as in a head‐and‐neck (HN) cancer patient case.^18^ However, the method was only validated in a few 6 MV photon beams for the purpose of proof of principles, and primary and scattered photons were not separated in that beam model. Hence, it is the first objective of this paper to apply the commissioning approach to our new analytical beam model in goMC, where the degrees of freedom are increased due to separation of primary and scattered sub‐sources. Moreover, we will further assess the validity of our analytical beam model and commissioning method on photon beams of different energies and flattening‐filter‐free (FFF) beams. In addition, some code modifications will be made to streamline the data preparation for commissioning.

The second motivation of this paper is to develop an effective sampling approach to incorporate plan parameters, e.g., beam angle, monitor unit (MU), and multi‐leaf collimator (MLC) configuration at control points, into MC dose calculation of a treatment plan. Although particle transport simulations may be performed within MLC, modeling its geometry and movements is not trivial^19^, ^20^ and adds additional computational burden.^21^, ^22^ Currently, no GPU‐based MC packages can support this function. Sampling source particles for each beam angle in accordance with the fluence map (FM) at that beam is a feasible alternative. The conventional way is to let each source particle carry the FM value of the position where the particle intersects the FM plane as its weight in dose calculation. This FM weighting approach has been widely employed in previous studies due to its simplicity.^7^, ^12^, ^23^, ^24^ However, because the MLC aperture at a control point is usually small, many source particles sampled from the beam model will hit the MLC leaves and carry small weights associated with MLC transmission. These particles contribute little to patient dose, indicating inefficient use of sampled particles in the computationally intensive MC simulations. This is not an issue for intensity modulated radiation therapy (IMRT) cases, as the FMs of IMRT plans are usually accumulated over the control points at a same beam angle to deal with the small MLC apertures at each individual control point. However, for volumetric modulated arc therapy (VMAT), the FM weighting approach becomes suboptimal. In this paper, we present an efficient source sampling approach to address this issue. It allows us to first sample the particle position on the FM plane based on the beam model, and then use the FM to bias the sampling of the control point to reduce the portion of those particles that would hit MLC leaves and contribute little to patient dose. Our experiments will demonstrate the accuracy and effectiveness of this method.

## 2 Methods and materials

### 2.A Automatic beam commissioning

The analytical beam model used in goMC was a field‐independent beam model, characterizing the particle distribution of the linac beam just above the secondary collimator. In our beam model, the phase‐space plane was partitioned into a set of phase‐space‐ring (PSR) sub‐sources.^17^ A PSR sub‐source is defined as a group of particles with the following characteristics: (1) spatially distributed in a narrow ring region, (2) within a small energy range, (3) same particle type (photon or electron), and (4) similar interaction history (primary or secondary). The particle intensity and direction distribution in each sub‐source were parameterized in analytical functions, by analyzing a reference phase‐space file. Our previous study has demonstrated the feasibility of using this analytical beam model to accurately represent the reference phase‐space file, and its efficiency benefit in GPU‐based MC simulations.^17^

However, manual adjustment of this analytical beam model to represent a real linac beam is impractical due to the large number of sub‐sources in the beam model; automation is therefore a desirable feature. Using the measured dose to adjust the optimal angular width parameters (i.e., the parameters that characterize the particle direction distribution within each sub‐source) of our beam model is challenging, since these parameters appear in exponential terms.^17^ Hence, we assumed that the angular width parameters fitted from a reference phase‐space file were accurate enough to model the local direction distribution of a real beam, and we only needed to finely tune the energy spectrum, the particle distribution, and the overall direction distribution to match the measured dose, by adjusting the relative intensity among the sub‐sources of different energies and locations. This assumption enabled us to model the commissioning as a linear problem, due to the linearity of the dose calculations. A multiplicative correction factor was hence introduced for commissioning. The dose distribution of a commissioned beam model is then a simple summation of the sub‐source doses weighted by their corresponding correction factors. These factors can be automatically adjusted by a numerical optimization algorithm that minimizes the differences between the actual measurement and the calculated dose. We have used this optimization‐based idea to commission a phase‐space‐let beam model previously, in which each sub‐source contained both the primary and scattered photons.^18^ In this study, we investigated the feasibility of this auto‐commissioning approach on our analytical beam model, which was a more challenging scenario with increased degrees of freedom because of the separated primary and scattered sub‐sources in the beam model.

#### 2.A.1 Pre‐calculating the dose contribution of each sub‐source

To determine these correction factors, we need to pre‐calculate the dose contribution of each sub‐source in water for open fields. In our previous study,^17^ our MC dose calculation code was repeatedly launched, sampling a certain amount of source particles from one sub‐source and transporting these particles in a water phantom to obtain the dose distribution of this sub‐source. The resulting 3D dose distribution was then post‐processed to extract the dose values at certain voxels (i.e., central axis depth dose and inline and cross‐line profiles at several depths) to be used for commissioning later. This workflow was time consuming mainly due to two reasons: (1) repeatedly initializing the MC code and transferring input data from CPU to GPU; (2) post‐processing a large data set of 3D dose distributions (e.g., a few hundred Gigabytes).

To address this issue and streamline the data preparation of commissioning, we have modified our dose calculation code to perform concurrent dose calculations for all the sub‐sources in one run of MC simulation, with separate storage for each sub‐source dose. The modifications are described as follows:

1. A 2D sub‐source dose array, denoted as matrix *A*, was allocated on GPU to record the dose contributions of all the sub‐sources for an open field. Each column of matrix *A* stored the doses of one sub‐source, but only at the voxels to be used in commissioning (i.e., central axis depth dose and inline and cross‐line profiles at several depths) instead of the entire 3D volume. Although a large number of sub‐sources were included in our analytical beam model, the size of this matrix was small enough relative to the limited GPU memory capacity, which made it possible to concurrently calculate the dose of all the sub‐sources in one run of MC simulation. The size of matrix *A* in our experiment was ˜24 MB, compared to ˜180 GB of the entire 3D dose volumes of all sub‐sources.
2. When the MC code was launched to calculate the dose for all the sub‐sources, the source particles were sampled from our analytical beam model and transported in a water phantom, as if conducting a typical dose calculation. The difference was that in this sub‐source dose calculation each source particle carried a sub‐source index denoting which sub‐source it was sampled from. Secondary particles generated during transport inherited the same index. When a particle deposited dose to a voxel of the water phantom that belonged to the commissioning data set, this dose value would be recorded into an entry of matrix *A*. The column index of this entry in matrix *A* was specified by the sub‐source index carried by the particle. The row index of this entry was quickly determined using a pre‐created row index look‐up table. For each voxel of the water phantom, this table stored its corresponding row index in matrix *A*. A negative value in the table denoted a voxel that would not be used in commissioning, hence dose depositions at this voxel would be ignored during MC simulation. This look‐up table was loaded onto GPU texture memory for fast access by GPU threads.

#### 2.A.2 Commissioning model

We conducted the commissioning process for photons and electrons separately, as the contaminant electrons in a photon beam have a small penetration depth and contribute dose only at shallow depth. We first adjusted the correction factors of all the photon PSR sub‐sources using the dose voxels at deep depths, where contributions from the electron sub‐sources were negligible. The commissioning problem for photon PSRs was mathematically formulated as

(1) $$x_{p}=min_{x_{p}\geq 0}||A_{1p}x_{p}-b_{1}|{|}^{2}.$$

As introduced in Section 2.A.1, matrix *A* is a matrix containing pre‐calculated sub‐source doses. Each column corresponds to the dose of a sub‐source, recorded at the voxels along the beam central axis and the inline and cross‐line profiles at several depths. *A*_1*p*_ in Eq.(1) is a submatrix of A with the columns corresponding to photon PSRs and the rows corresponding to the voxels at depths larger than the electron penetration depth (i.e., after the build‐up region). $b_{1}$ is a vector consisting of the measured data of a specific linac to be commissioned at those voxels. The $x_{p}$ vector contains the correction factor to be determined for each photon PSR. These correction factors should be non‐negative, since they will be multiplied to the original intensity of each PSR to reweigh their contributions in a beam.

Once the correction factors of the photon PSRs were determined, we then commissioned the electron PSRs to match the build‐up region. Note that the regions already commissioned in the last step were beyond the electron penetration depth, and hence not affected by this step. The electron PSRs within a same energy bin were grouped to form an “effective PSR,” and were adjusted together in our commissioning via a single correction factor. The sub‐source dose for an electron “effective PSR” was obtained by summing over the doses of all the electron PSRs within the corresponding energy bin. The purpose of this grouping strategy was to average out the relatively large statistical uncertainty in the dose distributions of the electron PSRs because of the very small amount of the contaminant electrons in a photon beam (~1% of the total particles).^18^ The electron commissioning model was formulated as follows:

(2) $$x_{e}=min_{x_{e}\geq 0}{||A_{2e}x_{e}+A_{2p}{\hat{x}}_{p}-b_{2}||}_{\wedge_{2}}^{2}.$$

Here, $x_{e}$ denotes a vector of the correction factor for each electron “effective PSR.” Each column of the matrix $A_{2e}$ corresponds to the dose for an effective PSR in the build‐up region. This matrix was obtained by fetching a submatrix of A with those columns for electron PSRs and rows for voxels in the build‐up region, and then summing over columns with the same energy bin. $A_{2p}{\hat{x}}_{p}$ denotes the total dose of the previously commissioned photon PSRs in the build‐up region. The $b_{2}$ term is the measured data in the same region. Both the problems in Eqs. (1) and (2) are a least square optimization problem, and gradient descent algorithm was used to solve them.

Compared to our previous approach developed for the phase‐space‐let beam model,^18^ this new commissioning method adapted to the analytical beam model has a number of new features. (1) The new commissioning model contains no regularization terms. The reason is that unlike the phase‐space‐let beam model, beam rotational symmetry is already implicitly imposed in the analytical beam model. This fact eliminates the need to impose the beam symmetry property onto commissioning through the regularization terms. (2) The energy spectrum of the electron sub‐sources is adjustable in this study, which was not allowed in our previous approach. Grouping the electron sub‐sources for each energy bin makes the commissioning robust against the uncertainty, while enabling the tuning of the energy spectrum of the contaminant electrons at the same time. (3) Unlike our previous study which only used the largest available field for commissioning, multiple field sizes need to be fed into the commissioning model to overcome the increased degrees of freedom due to separation of the primary and scattered photons in our analytical beam model.

We would like to mention that the penumbra regions were excluded from our commissioning model, since the sharp dose fall‐off at the penumbra would lead to large point‐wise dose difference and mislead the optimization. This does not mean fitting in the penumbra regions was totally neglected. Because the dose values at different voxels from a given sub‐source are spatially correlated, fitting the inner and outer beam regions can implicitly modify the dose in the penumbra region.

### 2.B FM‐based biased source sampling

When performing dose calculation for a specific VMAT treatment plan, three groups of information have to be considered: particle distribution in the beam model, MU distribution among different control points, and the spatial distribution of the MLC transmission factors at each control point corresponding to its MLC aperture. This is not a trivial task, if one would like to achieve a high efficiency. Generally speaking, each distribution can be incorporated either by altering particle weight or biasing source sampling according to the distribution. The latter is preferred because of its more effective use of source particles in MC simulations.

To achieve this, source sampling for VMAT treatment plan was conducted in two steps. The first step was to sample source particles from our field‐independent analytical beam model. A GPU‐friendly source sampling strategy has been designed for this step in our previous study.^17^ To be efficient, the sampling was only performed within or nearby the jaw open area. Once the source particles were sampled from our beam model, they followed a given distribution in terms of particle type, spatial location, direction and energy that was characterized by the model; the weight carried by each particle equaled to one. The second step was to incorporate the specific plan information into these sampled source particles. The FM value of a position on the 2D FM plane (i.e., a plane defined on the MLC upper surface using same coordinate system of our beam model) at a control point reflects not only the MU that the beam delivered at this control point, but also the MLC leaf open‐close status. It is preferable to bias source sampling based on FMs to reduce the portion of the sampled particles that would hit MLC leaves and contribute negligibly to patient dose.

In this study, we proposed a strategy to realize this biased sampling, referred to as the FM‐based biased sampling method hereon, employing the inverse sampling method.^25^ Specifically, let us denote the FM as $FM\left(x,y,k\right)$, with (x,y) being a position on the FM plane and k representing a control point. Given a source particle already sampled from our beam model, its position on the 2D FM plane is already determined. For this particle, the cumulative probability density function (CPDF) of the control points can be calculated as

(3) $$CPDF\left(m\right)=\frac{\sum_{k=0}^{m}FM\left(x,y,k\right)}{\sum_{k}FM\left(x,y,k\right)}.$$

This CPDF can be used to assign the control point index of the particle. For instance, after sampling a source particle from the beam model, we could generate a random number $\gamma \in \left[0,1\right]$ and search for the control point index k that satisfied $CPDF\left(k\right)\leq \gamma <CPDF\left(k+1\right)$. Then the source particle was rotated according to the beam configuration of this control point, and carried a weight equal to $\sum_{k}FM\left(x,y,k\right)$. However, searching for this control point index in the CPDF array is not preferred on GPUs, because of the sequential behavior and hence potential GPU thread divergence issues. This is particularly a concern for the treatment plans with a large number of control points. Hence, to quickly determine a control point index for each source particle, we pre‐created a numerical inverse look‐up table for each arc. This table listed the corresponding control point indices as a function of probability for each location on the FM plane. It was allocated on GPU texture memory for fast access by GPU threads. It can be seen that this look‐up table was independent from the beam model and was plan‐dependent, reflecting the MLC leaf sequence.

With our method, once a source particle is sampled from the distribution of a beam model, it will have a high probability to be assigned to the control point that not only has a relatively large MU but also has the MLC leaves open at the particle's position on the FM plane. It is this effect that makes our approach more efficient, requiring fewer source particles to achieve a targeted uncertainty level.

### 2.C Materials

Six analytical beam models were built for Varian TrueBeam (Varian Medical System, Palo Alto, CA, USA) photon beams including 6 MV, 10 MV, 15 MV, 18 MV beams, and two FFF beams of 6 MV and 10 MV. These six beam models were built by analyzing the corresponding reference phase‐space files.^17^ A beam model consists of 2400 PSR sub‐sources, given 40 rings, 20 energy bins, and 3 PSR types (i.e., primary photon, scattered photon, and contaminant electron) we used to partition the phase‐space. Our auto‐commissioning approach present in this paper was employed to automatically commission these models to the Varian TrueBeam beam used in our institution. For each beam, the depth dose and the inline and cross‐line profiles at depths of 5 cm, 10 cm, 20 cm, and $d_{max}$ (i.e., the depth where the dose reached its maximum value) were acquired for multiple open fields with field sizes of 2 × 2 cm^2^, 5 × 5 cm^2^, 10 × 10 cm^2^, 20 × 20 cm^2^, and 40 × 40 cm^2^. A diode detector was used for the 2 × 2 cm^2^ small field; a CC13 cylindrical ion chamber was used for data acquisition of other four fields. The measured doses of 40 × 40 cm^2^, 10 × 10 cm^2^, and 2 × 2 cm^2^ fields were used for commissioning. The corresponding sub‐source dose set (i.e., matrix *A*) were pre‐calculated for use in the commissioning model. After commissioning, the depth dose, lateral profiles, and output factors for all the five fields (including the two fields not used for commissioning) were calculated using the commissioned beam models and our dose engine goMC, and compared with the measurements to validate the accuracy of our commissioning. For the purpose of comparison, we also calculated the corresponding dose using the uncommissioned beam models, referred as to the reference beam model hereon.

A typical VMAT HN patient case was used as an example to demonstrate the practicality and efficiency gain of our FM‐based biased source sampling method. This treatment plan had two arcs, each with 178 control points. MC dose calculations were performed for this VMAT case, using our proposed biased sampling method and the FM weighting method, respectively. The overall efficiency of the dose calculation was tested and compared among these two methods.

We also investigated the overall accuracy of our goMC dose engine with the commissioned beam model and the new source sampling method for clinical use. The dose distribution of a VMAT prostate patient case with a 10 MV FFF beam was calculated, and compared with the dose calculated in a commercial treatment planning system (TPS) used in our institution, i.e., Varian Eclipse TPS (Varian Medical System, Palo Alto, CA, USA). In this study, all MC simulations were performed on an NVidia GeForce GTX Titan Black GPU card (NVidia Corporation, Santa Clara, CA, USA).

## 3 Results

### 3.A Automatic beam commissioning

With the improved sub‐source dose calculation method introduced in Section 2.A.2, it took ~6 h on a Titan Black GPU card to pre‐calculate the sub‐source doses for commissioning a beam model. The time saving due to our code modification was estimated to be ~18 h. When launching a MC dose calculation, the time spent on code initialization was about ~6 s. The time to post process a 3D dose volume was about ~3 s. Given three open fields used in our commissioning and 2400 PSR sub‐sources contained in our beam model, ~12 h should be saved by avoiding the repeated code initialization for each sub‐source, and ~6 h saved by eliminating the post‐processing procedure on the 3D dose volumes of the sub‐sources. We would like to mention that even with our improvement, the computation time for the sub‐source dose calculation was still high. That is, due to the required large number of source particles to generate matrix *A* with a very low statistical uncertainty (~0.2% on average) in order to reduce the effect of the uncertainty on commissioning. Nevertheless, this sub‐source dose calculation step needs to be performed only once. Once the sub‐source doses are available, they can be reused each time when we commission with respect to a linac beam. The optimization problems in the commissioning model were solved in ~20 s for each linac beam, using Matlab (MathWorks, Natick, MA). Due to page limitations, we will only present the commissioning results of three representative beams (i.e., the 6 MV and 15 MV photon beams and the 10 MV FFF photon beam). Similar levels of commissioning accuracy were also achieved for the other three beams.

#### 3.A.1 6 MV photon beam with flattening filter (FF)

**Figure 1:**
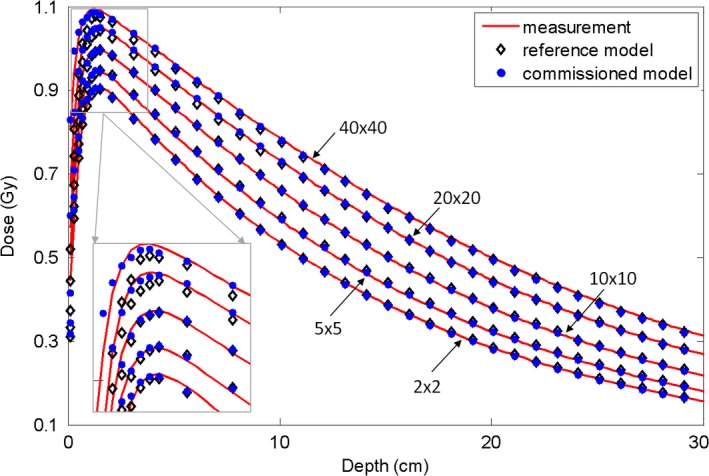
Comparison of depth dose curves for the 6 MV FF photon beam between the measurement data (solid line), data calculated with the reference analytical beam model (open diamond), and data calculated with the commissioned model (solid circle). Five open fields (40 × 40 cm^2^ 20 × 20 cm^2^, 10 × 10 cm^2^, 5 × 5 cm^2^, 2 × 2 cm^2^) with 100 cm SSD are shown. The region close to the $d_{max}$ is enlarged. The dose points after the $d_{max}$ were downsampled for clear display.

**Figure 2:**
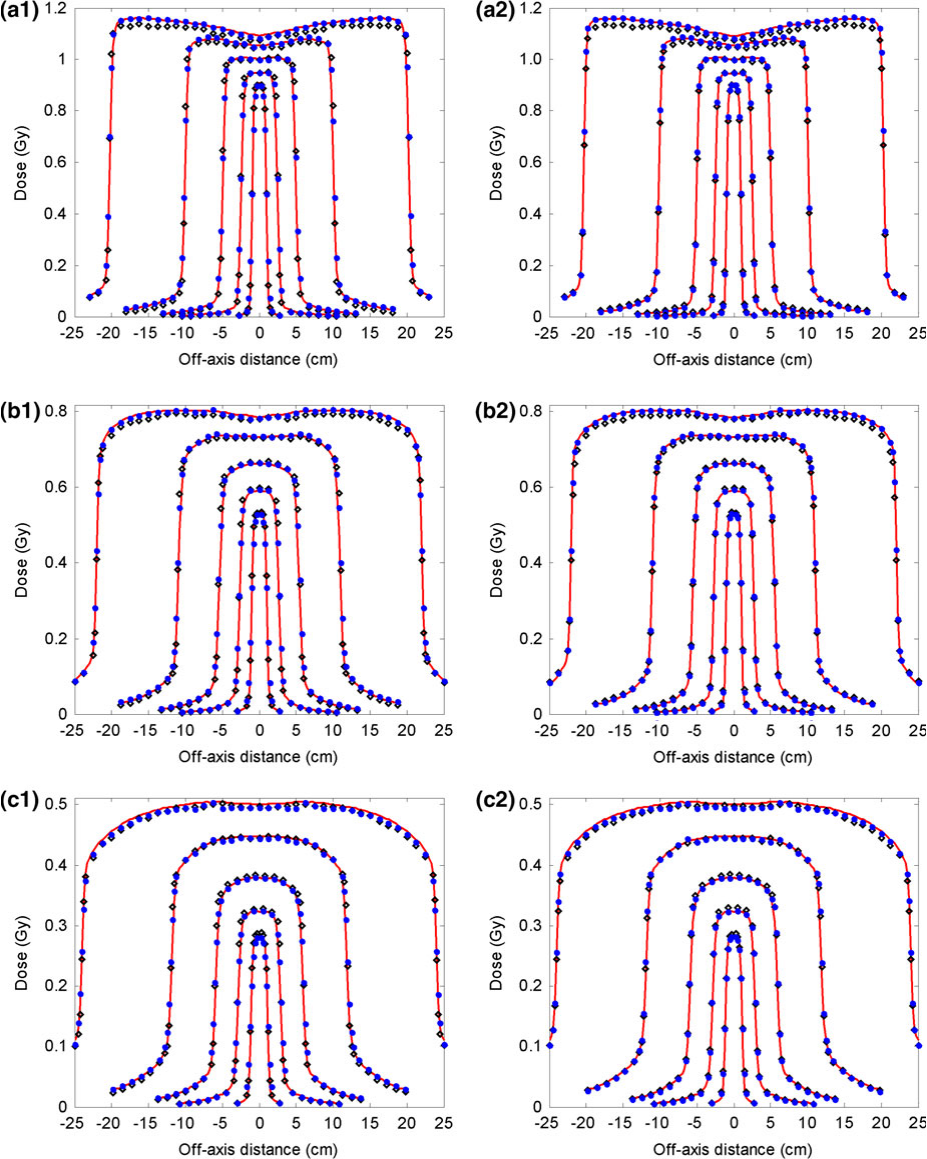
Comparison of inline and cross‐line lateral dose profiles of the 6 MV FF photon beam between measurement data (solid line), data calculated with the reference analytical beam model (open diamond), and data calculated with our commissioned model (solid circle). Five open fields (40 × 40 cm^2^, 20 × 20 cm^2^, 10 × 10 cm^2^, 5 × 5 cm^2^, 2 × 2 cm^2^) with 100 cm SSD are shown. (a1–c1): Inline dose profiles of these five open fields at depths of 1.5 cm, 10 cm, and 20 cm, respectively; (a2–c2): Corresponding cross‐line dose $d_{max}$ profiles. The dose points at inner and outer beam regions were downsampled for clear display.

Using the commissioned 6 MV FF photon beam model, the dose distributions were calculated for five open fields (40 × 40, 20 × 20, 10 × 10, 5 × 5, and 2 × 2 cm^2^) with a 100 cm SSD. The calculated depth doses and the inline and cross‐line lateral dose profiles at three depths (1.5 cm, 10 cm, and 20 cm) were compared with the measurement data, and with the dose calculated using the reference beam model (Figs. 1 and 2). Obvious discrepancies in the depth dose curves were observed between the reference model and the measurements, particularly obvious for the build‐up region of the large fields (Fig. 1). This implied that the energy spectrum of the reference beam model was different from that of the actual linac beam. Using depth dose and profiles at different depths, the energy spectrum of the beam model was finely tuned during commissioning via adjusting the correction factors of the sub‐sources of different energies. As a consequence, our commissioning results better matched the measured depth dose in the build‐up region, as shown in Fig. 1. Relatively large dose discrepancies between the reference model and the measurement were found for the 40 × 40 and 20 × 20 cm^2^ fields at the inner beam region in both inline and cross‐line directions (Fig. 2). Obvious dose discrepancies were also noted at the outer beam region of the inline dose profiles for the 20 × 20 cm^2^ field. By reweighting the sub‐sources in our analytical beam model, the commissioned model better agreed with the measurement in terms of lateral profiles.

These results were further quantitatively evaluated using region‐specific metrics as suggested by AAPM task group 53.^26^ Specifically, the root‐mean‐square (RMS) difference and maximum difference were calculated for the regions with relatively low‐dose gradients, including the depth dose after build‐up and the lateral dose profiles at inner and outer beam regions. These two metrics were calculated as:

(4) $$RMS\left(\%\right)=\frac{1}{D_{max}^{m}}\sqrt{\frac{1}{N}\sum_{i=1}^{N}{\left(D_{i}^{c}-D_{i}^{m}\right)}^{2}},$$

(5) $$Max\left(\%\right)=\frac{1}{D_{max}^{m}}max_{i}\left|D_{i}^{c}-D_{i}^{m}\right|.$$

$D_{i}^{c}$ denotes the dose value of the $i_{th}$ dose point calculated with either the reference analytical beam model or the commissioned model. $D_{i}^{m}$ is the corresponding measurement data, considered as the ground truth for comparison. The $D_{max}^{m}$ parameter denotes the measured depth dose at $d_{max}$. For high‐gradient regions, including the depth dose at the build‐up region and lateral dose profiles at the penumbra region, we employed the distance‐to‐agreement (DTA) for evaluation. The DTA at a spatial location x is defined as the minimum distance s=|x−y| so that $D^{c}\left(y\right)=D^{m}\left(x\right)$.

The quantitative evaluation results of the depth dose and the evaluation results of the inline dose profiles are presented in Tables 1 and 2, respectively. The major improvement was found at the inner beam region. Our commissioned 6 MV FF photon beam model improved the RMS and maximum difference at the inner beam region from 0.31–2.01% to 0.01–0.78% and from 0.47–3.22% to 0.15–3.21%, respectively. Improvements were also noted at the outer beam region, with RMS from 0.20–1.25% to 0.10–0.51% and maximum difference from 0.27–1.95% to 0.15–0.75%, respectively. A similar level of improvement was observed for the cross‐line dose profiles (data not shown). The improvements on the depth dose in terms of DTA at the build‐up region and RMS after build‐up region were relatively small.

**Table 1:** Quantitative evaluation results of depth dose curves for the 6 MV FF photon beam compared with measurement data. $D_{ref}$: dose calculated with the reference analytical source model; $D_{com}$: dose calculated with the commissioned model

Field size (cm^2^)	Build‐up region				Region after build‐up			
	Average DTA (cm)		Maximum DTA (cm)		RMS (%)		Max (%)	
	$D_{\mathrm{ref}}$	$D_{\mathrm{com}}$	$D_{\mathrm{ref}}$	$D_{\mathrm{com}}$	$D_{\mathrm{ref}}$	$D_{\mathrm{com}}$	$D_{\mathrm{ref}}$	$D_{\mathrm{com}}$
40×40	0.28	0.12	0.47	0.37	0.66	0.48	1.55	0.90
20×20	0.18	0.07	0.36	0.20	0.43	0.19	1.59	0.58
10×10	0.10	0.06	0.13	0.11	0.32	0.21	0.64	0.59
5×5	0.07	0.04	0.12	0.11	0.67	0.37	1.13	0.77
2×2	0.04	0.04	0.09	0.10	0.43	0.48	0.87	0.93

**Table 2:** Quantitative evaluation results of inline lateral dose profiles for the 6 MV FF photon beam compared with the measurement data. $D_{ref}$: dose calculated with the reference analytical source model; *D*_*com*_: dose calculated with our commissioned model

Field size (cm^2^)	Depth (cm)		Penumbra		Inner beam		Outer beam	
			Average DTA (cm)	Maximum DTA (cm)	RMS (%)	Max (%)	RMS (%)	Max (%)
40×40	1.5	$D_{ref}$	0.08	0.12	2.01	2.88	1.03	1.62
		$D_{com}$	0.07	0.11	0.43	1.23	0.30	0.49
	10	$D_{ref}$	0.04	0.13	1.11	1.95	0.63	0.75
		$D_{com}$	0.03	0.07	0.32	0.80	0.32	0.40
	20	$D_{ref}$	0.04	0.08	0.66	1.30	0.97	1.03
		$D_{com}$	0.04	0.06	0.55	1.03	0.51	0.66
20×20	1.5	$D_{ref}$	0.08	0.12	1.16	3.22	1.25	1.48
		$D_{com}$	0.07	0.11	0.78	3.21	0.35	0.53
	10	$D_{ref}$	0.10	0.17	0.45	1.22	0.63	0.70
		$D_{com}$	0.08	0.12	0.43	0.88	0.32	0.39
	20	$D_{ref}$	0.10	0.18	0.31	0.64	0.43	0.52
		$D_{com}$	0.09	0.13	0.31	0.56	0.10	0.15
10×10	1.5	$D_{ref}$	0.08	0.14	0.60	1.09	0.86	1.58
		$D_{com}$	0.08	0.11	0.44	1.09	0.28	0.54
	10	$D_{ref}$	0.10	0.22	0.71	1.28	0.41	0.65
		$D_{com}$	0.09	0.12	0.46	0.90	0.28	0.75
	20	$D_{ref}$	0.10	0.21	0.52	1.08	0.25	0.58
		$D_{com}$	0.09	0.13	0.06	0.15	0.14	0.61
5×5	1.5	$D_{ref}$	0.08	0.13	0.60	1.38	0.60	1.14
		$D_{com}$	0.07	0.10	0.55	1.43	0.29	0.70
	10	$D_{ref}$	0.08	0.14	1.19	2.27	0.30	0.46
		$D_{com}$	0.08	0.11	0.67	1.36	0.12	0.36
	20	$D_{ref}$	0.09	0.14	0.70	1.46	0.20	0.27
		$D_{com}$	0.08	0.11	0.13	0.30	0.12	0.31
2×2	1.5	$D_{ref}$	0.07	0.11	0.76	0.92	0.20	0.28
		$D_{com}$	0.07	0.12	0.65	0.71	0.21	0.30
	10	$D_{ref}$	0.08	0.28	0.58	1.14	0.71	1.68
		$D_{com}$	0.10	0.31	0.53	1.07	0.16	0.32
	20	$D_{ref}$	0.11	0.31	0.40	0.47	0.84	1.95
		$D_{com}$	0.12	0.34	0.33	0.43	0.17	0.39

**Figure 3:**
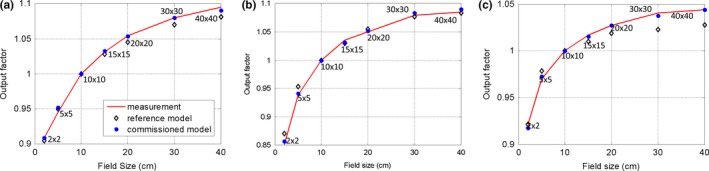
Output factor comparison between measurement data, the reference analytical source model, and the commissioned model. (a) 6 MV FF photon beam; (b) 15 MV FF photon beam; (c) 10 MV FFF photon beam.

The output factors of our commissioned source model, which were defined at 100 cm SSD and *d*_*max*_ depth, were also calculated and compared with the measurement and reference source model, as shown in Fig. 3(a). After commissioning, the maximum absolute difference was improved from 1.29 to 0.64% as compared with the measurement data. The average difference was improved from 0.61 to 0.17%.

#### 3.A.2 15 MV photon beam with flattening filter (FF)

**Figure 4:**
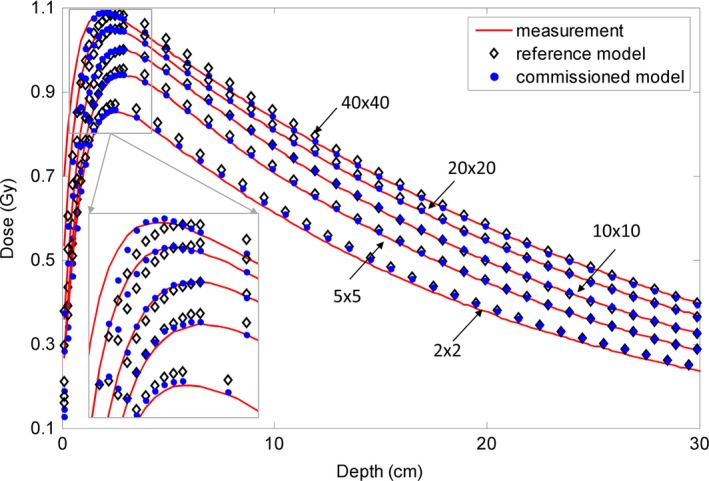
Comparison results of depth dose curves for the 15 MV FF photon beam between measurement data (solid line), data calculated with the reference analytical beam model (open diamond), and data calculated with the commissioned model (solid circle). Five open fields (40 × 40 cm^2^, 20 × 20 cm^2^, 10 × 10 cm^2^, 5 × 5 cm^2^, 2 × 2 cm^2^) with 100 cm SSD are shown. The region close to $d_{max}$ are zoomed‐in. The dose points after $d_{max}$ were downsampled for clear display.

**Figure 5:**
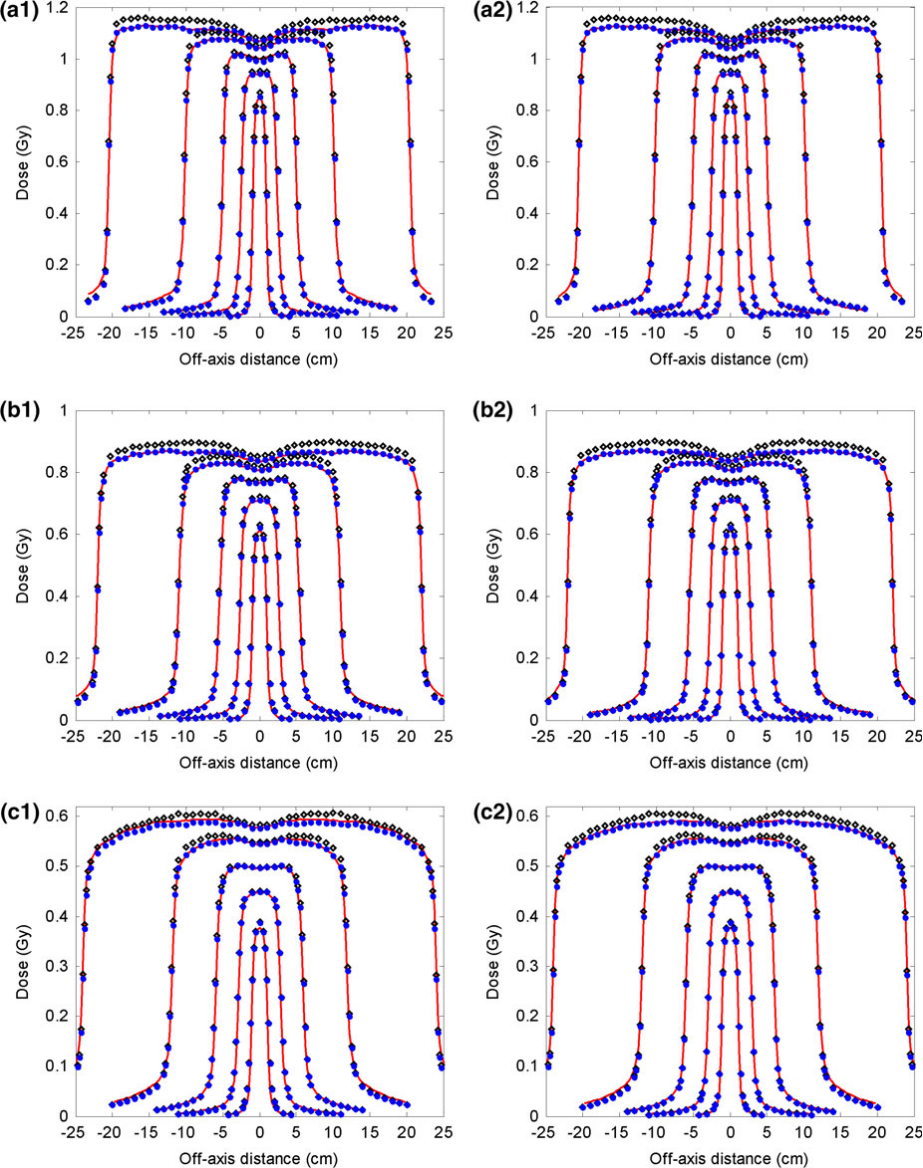
Comparison results of inline and cross‐line lateral dose profiles for the 15 MV FF photon beam between measurement data (solid line), data calculated with the reference analytical beam model (open diamond), and data calculated with the commissioned model (solid circle). Five open fields (40 × 40 cm^2^, 20 × 20 cm^2^, 10 × 10 cm^2^, 5 × 5 cm^2^, 2 × 2 cm^2^) with 100 cm SSD are compared here. (a1–c1): Inline dose profiles of those open fields at depths of 2.8 cm, 10 cm and 20 cm, respectively; (a2–c2): Corresponding cross‐line dose profiles. The dose points at the inner and outer beam regions were downsampled for clear display.

Comparisons of depth dose and inline and cross‐line lateral dose profiles at three depths (2.8 cm, 10 cm, and 20 cm) for the 15 MV FF photon beam are shown in Figs. 4 and 5. Discrepancies were observed between the reference model and the measurements at the build‐up region for the depth dose. Large differences were also noted at the inner beam region on both inline and cross‐line directions for large fields (i.e., 40 × 40 and 20 × 20 cm^2^ fields) and small fields (i.e., 20 × 20 cm^2^ field). After commissioning, these discrepancies were significantly reduced. Quantitative comparison results of the depth dose curves and the inline lateral dose profiles are displayed in Tables 3 and 4, respectively. For the depth dose curves, the average DTA was reduced from 0.12–0.38 cm to 0.06–0.20 cm, and the maximum DTA from 0.20–0.50 cm to 0.17–0.35 cm at the build‐up region; the RMS and maximum difference were reduced from 0.42–1.96% to 0.22–1.27% and from 1.02–3.04% to 0.46–1.61%, respectively, at the region after build‐up. RMS and maximum differences at the inner beam region were improved from 0.40–2.77% to 0.21–1.02% and from 1.01–4.52% to 0.61–1.95%, respectively. The calculated output factors are shown in Fig. 3(b). The maximum absolute differences of the output factors and the average difference improved from 1.55 to 0.68% and from 0.76 to 0.34%, respectively.

**Table 3:** Quantitative evaluation results of depth dose curves for the 15 MV photon beam compared with the measurement data. $D_{ref}$: dose calculated with the reference analytical source model; *D*_*com*_: dose calculated with our commissioned model

Field size (cm^2^)	Build‐up region				Region after build‐up			
	Average DTA (cm)		Maximum DTA (cm)		RMS (%)		Max (%)	
	$D_{\mathrm{ref}}$	$D_{\mathrm{com}}$	$D_{\mathrm{ref}}$	$D_{\mathrm{com}}$	$D_{\mathrm{ref}}$	$D_{\mathrm{com}}$	$D_{\mathrm{ref}}$	$D_{\mathrm{com}}$
40×40	0.38	0.20	0.49	0.35	0.70	0.54	1.87	0.75
20×20	0.23	0.15	0.29	0.25	0.99	0.22	2.15	0.46
10×10	0.12	0.06	0.20	0.17	0.42	0.36	1.02	0.62
5×5	0.12	0.12	0.50	0.32	1.05	0.32	2.02	0.57
2×2	0.13	0.09	0.42	0.20	1.96	1.27	3.04	1.61

**Table 4:** Quantitative evaluation results of inline lateral dose profiles for the 15 MV photon beam compared with the measurement data. *D*_*ref*_: dose calculated with the reference analytical source model; *D*_*com*_: dose calculated with our commissioned model

Field size (cm^2^)	Depth (cm)		Penumbra		Inner beam		Outer beam	
			Average DTA (cm)	Maximum DTA (cm)	RMS (%)	Max (%)	RMS (%)	Max (%)
40×40	2.1	$D_{ref}$	0.08	0.14	2.77	4.52	2.15	2.74
		$D_{com}$	0.07	0.16	0.38	1.54	2.44	3.09
	10	$D_{ref}$	0.09	0.17	2.30	3.31	0.65	1.32
		$D_{com}$	0.07	0.13	0.26	0.92	0.80	1.52
	20	$D_{ref}$	0.05	0.12	1.10	1.87	0.59	0.99
		$D_{com}$	0.08	0.15	0.21	0.61	0.87	1.40
20×20	2.1	$D_{ref}$	0.07	0.13	2.14	4.31	1.18	2.64
		$D_{com}$	0.05	0.14	0.37	0.77	1.10	2.61
	10	$D_{ref}$	0.08	0.21	2.02	3.56	0.68	1.35
		$D_{com}$	0.05	0.13	0.28	0.97	0.80	1.61
	20	$D_{ref}$	0.07	0.16	0.85	1.72	0.76	1.18
		$D_{com}$	0.05	0.15	0.33	0.75	0.88	1.42
10×10	1.5	$D_{ref}$	0.07	0.15	0.93	2.44	0.86	2.12
		$D_{com}$	0.05	0.14	0.61	1.50	0.68	1.81
	10	$D_{ref}$	0.06	0.10	0.74	1.80	0.43	1.12
		$D_{com}$	0.03	0.09	0.45	0.91	0.38	1.03
	20	$D_{ref}$	0.04	0.08	0.40	1.06	0.32	0.70
		$D_{com}$	0.02	0.07	0.35	0.68	0.29	0.67
5×5	1.5	$D_{ref}$	0.05	0.07	1.40	2.12	0.75	1.72
		$D_{com}$	0.04	0.07	0.53	1.31	0.73	1.60
	10	$D_{ref}$	0.04	0.07	1.41	2.40	0.81	2.76
		$D_{com}$	0.04	0.06	0.80	1.95	0.84	2.85
	20	$D_{ref}$	0.03	0.05	0.51	1.01	0.28	0.55
		$D_{com}$	0.03	0.05	0.52	1.12	0.30	0.59
2×2	1.5	$D_{ref}$	0.06	0.11	1.94	2.48	1.10	3.48
		$D_{com}$	0.06	0.12	0.57	0.82	1.15	3.69
	10	$D_{ref}$	0.06	0.11	1.90	2.18	1.19	3.95
		$D_{com}$	0.06	0.11	0.86	1.19	1.16	3.91
	20	$D_{ref}$	0.08	0.14	1.18	1.40	0.52	1.62
		$D_{com}$	0.07	0.14	1.02	1.34	0.48	1.53

#### 3.A.3 10 MV FFF photon beam

**Figure 6:**
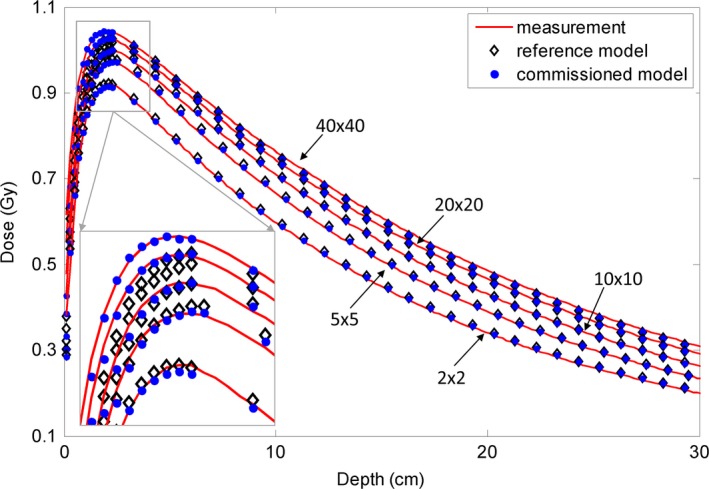
Comparison of depth dose curves for the 10 MV FFF photon beam between measurement data (solid line), data calculated with the reference analytical beam model (open diamond), and data calculated with the commissioned model (solid circle). Five open fields (40 × 40 cm^2^, 20 × 20 cm^2^, 10 × 10 cm^2^, 5 × 5 cm^2^, 2 × 2 cm^2^) with 100 cm SSD are compared here. The region close to $d_{max}$ is zoomed‐in. The dose points after $d_{max}$ were downsampled for clear display. The dose points at the inner and outer beam regions were downsampled for clear display.

**Figure 7:**
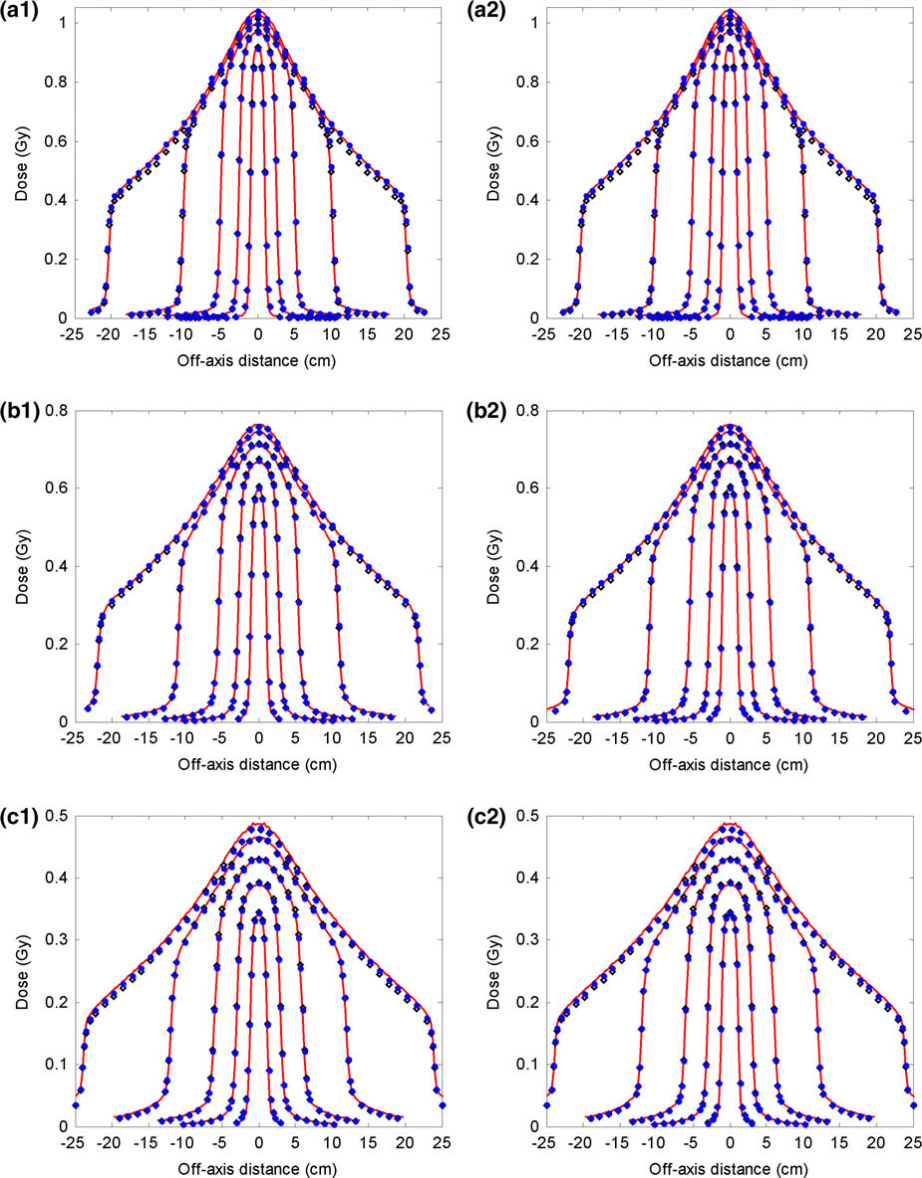
Comparisons of the inline and cross‐line lateral dose profiles for the 10 MV FFF photon beam between measurement data (solid line), data calculated with the reference analytical source model (open diamond), and data calculated with the commissioned model (solid circle). Five open fields (40 × 40 cm^2^, 20 × 20 cm^2^, 10 × 10 cm^2^, 5 × 5 cm^2^, 2 × 2 cm^2^) with SSD of 100 cm are compared here. (a1–c1): Inline dose profiles of those open fields at depths of 2.1 cm, 10 cm, and 20 cm, respectively. (a2–c2): Corresponding cross‐line dose profiles.

The depth doses for the 10 MV FFF photon beam and inline and cross‐line lateral dose profiles at three depths (2.1 cm, 10 cm, and 20 cm) are shown in Figs. 6 and 7. For the depth dose, large discrepancies were observed between the reference model and the measurements at the build‐up region for the two largest fields. For the lateral dose profiles, obvious discrepancies were detected for the largest field. The commissioned beam achieved a better agreement with the measurement data. Quantitative comparative results are summarized in Tables 5 and 6. For the depth dose, the average DTA was reduced from 0.04–0.29 cm to 0.04–0.07 cm and the maximum DTA from 0.09–0.48 cm to 0.10–0.15 cm at the build‐up region; RMS and maximum differences were reduced from 0.34–0.75% to 0.21–0.54% and from 0.87–1.87% to 0.47–0.88%, respectively, at the region after build‐up. RMS and maximum differences at the inner beam region were improved from 0.28–2.12% to 0.14–0.80% and 0.53–3.00% to 0.22–1.78%, respectively. Calculated output factors for this beam are shown in Fig. 3(c). The maximum absolute difference of the output factors and the average difference improved from 1.70 to 0.37% and from 0.80 to 0.14%, respectively.

**Table 5:** Quantitative evaluation results of depth dose curves for the 10 MV FFF photon beam compared with the measurement data. $D_{ref}$; dose calculated with the reference analytical source model; *D*_*com*_: dose calculated with our commissioned model

Field size (cm^2^)	Build‐up region				Region after build‐up			
	Average DTA (cm)		Maximum DTA (cm)		RMS (%)		Max (%)	
	$D_{\mathrm{ref}}$	$D_{\mathrm{com}}$	$D_{\mathrm{ref}}$	$D_{\mathrm{com}}$	$D_{\mathrm{ref}}$	$D_{\mathrm{com}}$	$D_{\mathrm{ref}}$	$D_{\mathrm{com}}$
40×40	0.29	0.05	0.48	0.15	0.70	0.54	1.87	0.75
20×20	0.23	0.06	0.45	0.13	0.34	0.24	1.18	0.47
10×10	0.11	0.07	0.15	0.12	0.37	0.21	0.93	0.49
5×5	0.04	0.05	0.10	0.10	0.75	0.41	1.52	0.88
2×2	0.05	0.04	0.10	0.11	0.62	0.42	0.87	0.88

**Table 6:** Quantitative evaluation results of inline lateral dose profiles for the 10 MV FFF photon beam compared with the measurement data. $D_{ref}$: dose calculated with the reference analytical source model; *D*_*com*_: dose calculated with our commissioned model

Field size (cm^2^)	Depth (cm)		Penumbra		Inner beam		Outer beam	
			Average DTA (cm)	Maximum DTA (cm)	RMS (%)	Max (%)	RMS (%)	Max (%)
40×40	2.1	$D_{ref}$	0.11	0.27	2.12	3.00	0.79	0.87
		$D_{com}$	0.09	0.19	0.64	1.78	0.30	0.36
	10	$D_{ref}$	0.05	0.14	0.73	1.35	1.06	1.23
		$D_{com}$	0.02	0.05	0.39	1.23	0.97	1.08
	20	$D_{ref}$	0.04	0.08	0.87	1.22	1.38	1.49
		$D_{com}$	0.04	0.05	0.73	1.56	1.30	1.45
20×20	2.1	$D_{ref}$	0.04	0.08	1.31	2.23	0.68	0.97
		$D_{com}$	0.07	0.11	0.76	1.57	0.25	0.31
	10	$D_{ref}$	0.04	0.10	0.38	0.77	0.48	0.75
		$D_{com}$	0.04	0.07	0.37	0.70	0.33	0.50
	20	$D_{ref}$	0.04	0.12	0.28	0.60	0.37	0.52
		$D_{com}$	0.04	0.08	0.50	1.02	0.26	0.36
10×10	1.5	$D_{ref}$	0.04	0.08	0.64	1.46	0.52	0.65
		$D_{com}$	0.04	0.09	0.60	1.43	0.30	0.40
	10	$D_{ref}$	0.04	0.07	0.75	1.73	0.35	0.53
		$D_{com}$	0.04	0.06	0.45	1.27	0.27	0.46
	20	$D_{ref}$	0.06	0.12	0.34	0.67	0.26	0.38
		$D_{com}$	0.04	0.11	0.28	0.72	0.21	0.34
5×5	1.5	$D_{ref}$	0.03	0.06	0.60	1.07	0.25	0.35
		$D_{com}$	0.02	0.06	0.40	0.79	0.20	0.28
	10	$D_{ref}$	0.04	0.06	1.27	1.59	0.16	0.32
		$D_{com}$	0.04	0.07	0.80	1.06	0.16	0.35
	20	$D_{ref}$	0.02	0.05	0.76	0.99	0.07	0.14
		$D_{com}$	0.02	0.05	0.53	0.72	0.08	0.14
2×2	1.5	$D_{ref}$	0.07	0.12	0.56	0.83	1.15	2.11
		$D_{com}$	0.07	0.12	0.14	0.22	1.17	2.15
	10	$D_{ref}$	0.06	0.10	0.38	0.63	1.28	3.10
		$D_{com}$	0.06	0.10	0.36	0.66	1.28	3.11
	20	$D_{ref}$	0.07	0.13	0.31	0.53	1.23	2.97
		$D_{com}$	0.06	0.13	0.20	0.37	1.23	2.96

Although in an actual clinical setting the measured data at all field sizes were for the purpose of beam commissioning, our results show that using three field sizes (40 × 40, 10 × 10, and 2 × 2 cm^2^) to commission our beam model already led to a sufficient accuracy for other different field sizes. That is because our analytical beam model was derived by analyzing a reference phase‐space file, and hence already a good approximation of the linac beam. The commissioning was to further finely tune the beam model for each specific clinical beam. We expect that using more field sizes will improve the commissioning result, but probably incrementally.

### 3.B Efficient source sampling

**Figure 8:**
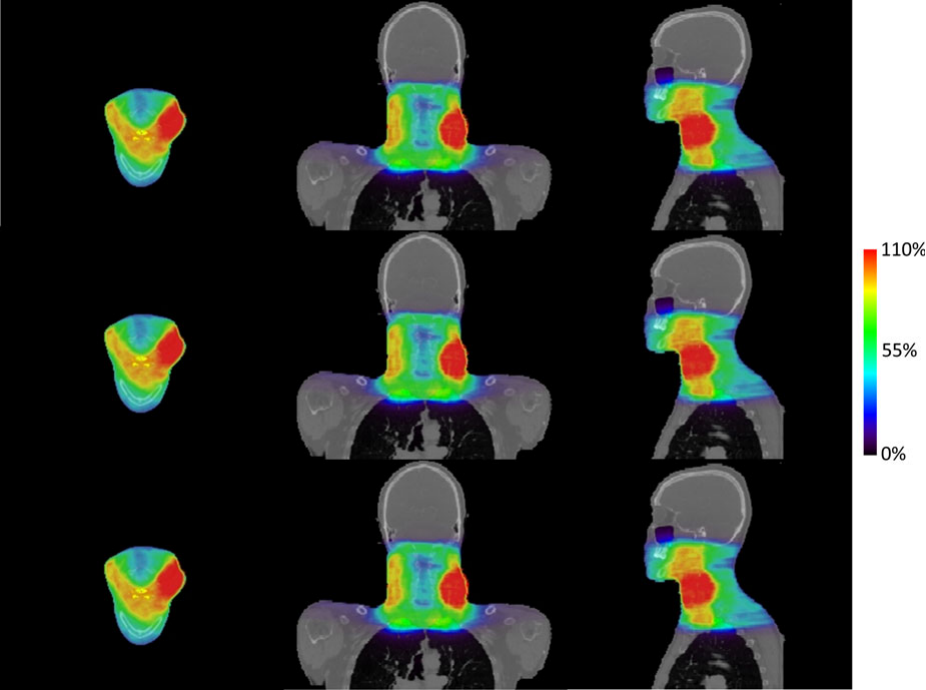
The dose distributions calculated for the HN VMAT patient case, shown in transverse, coronal, and sagittal views. The two rows correspond to the FM weighting method and our FM‐based biased sampling method, respectively. These two dose distributions have ~0.25% average statistical uncertainty, achieved when using 6.00 × 10^10^ source particles for the FM weighting method and 2.16 × 10^10^ for our method. The third row shows the absolute difference between these two doses. Note that a different color map scale was used to better display this difference.

Since our FM‐based biased source sampling method and the FM weighting method model the same physical process, they are expected to give us the same result at the limit of zero statistical uncertainty. To demonstrate this first, we calculated the dose distributions of a VMAT HN patient case with these two methods, respectively, both achieving 0.25% average statistical uncertainty (relative to the prescription dose). The doses are shown in Fig. 8. The average absolute difference between these two doses was 0.64% (relative to the prescription dose). We also performed 3D Gamma‐index test, and achieved 99.78% passing rate for the 2%‐2 mm criterion and 94.87% for the 1%‐1 mm criterion.

We then investigated the overall efficiency of MC dose calculations on this VMAT case when using these two source sampling methods. Since the computation time of MC simulation was associated with the achieved statistical uncertainty, the same uncertainty level was achieved by these methods to ensure fair comparison. As listed in Table 7, to achieve 0.25% statistical uncertainty, our method required 2.16 × 10^10^ source particles, compared to 6.00 × 10^10^ particles needed by the FM weighting method. The reduction factor on the number of the required source particles was ~2.8. The same phenomenon was observed when changing the targeted statistical uncertainty to 0.99%. This fact led to a comparable speed‐up factor on the dose calculations. The dose calculation for this patient case was finished in 51.5 s when using our new method, with 0.99% uncertainty achieved. The corresponding computation time for the FM weighting method was 150.9 s. These results have well demonstrated the efficiency gain of our biased sampling method.

**Table 7:** Efficiency comparison among the two methods to incorporate FM into MC dose calculation for the HN VMAT case

	FM weighting	FM‐based biased sampling
Statistical uncertainty (%)	0.26	0.25
No. of source particles (10^9^)	60.00	21.60
Computational time (s)	2274.8	823.9
Statistical uncertainty (%)	0.98	0.99
No. of source particles (10^9^)	3.75	1.35
Computational time (s)	150.9	51.5

To better understand how our method outperformed the FM weighting method in terms of efficiency, we recorded the number of the sampled “less useful” source particles (i.e., the source particles that hit the MLC leaves). Among 1.35 × 10^9^ source particles sampled from the beam model, 4.01 × 10^8^ particles were found to be “less useful” particles when using the FM weighting method, accounting for 29.70% of the sampled particles. In contrast, when sampling the same amount of source particles with our method, 1.46 × 10^8^ “less useful” particles were found, accounting for 10.81% of the total amount. These numbers demonstrated that our FM‐based biased source sampling could effectively reduce the portion of the “less useful” source particles, which hence lead to an overall efficiency gain in MC dose calculations.

### 3.C Comparison with commercial treatment planning system

**Figure 9:**
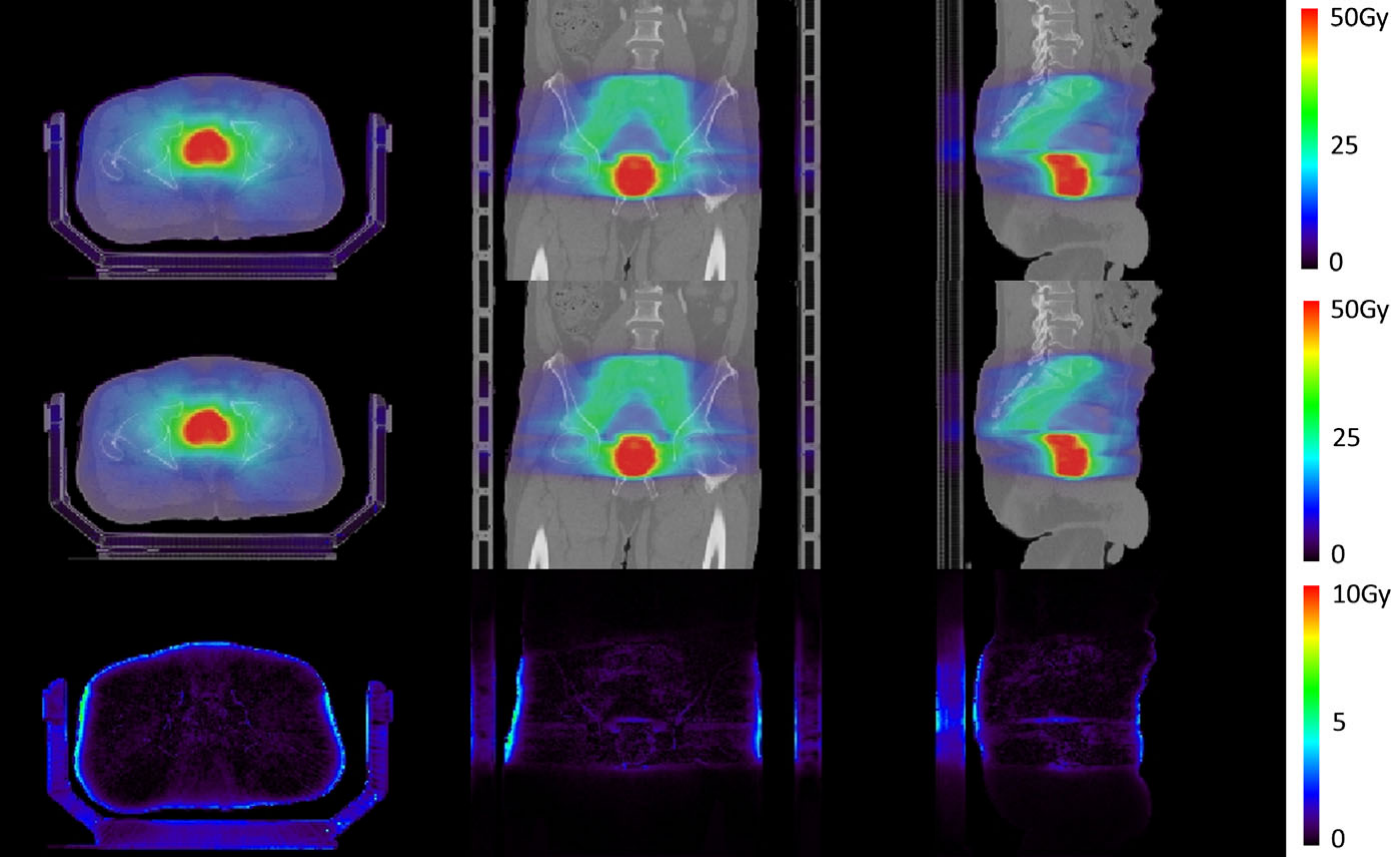
Dose distributions calculated for a prostate VMAT patient case with 10 MV FFF photon beam. Top: dose calculated in a commercial TPS (Varian Eclipse); Middle: dose calculated in our goMC dose engine with the commissioned beam model; Bottom: absolute dose difference between these two dose distributions. Note that a different color map scale was used to better display this difference.

**Figure 10:**
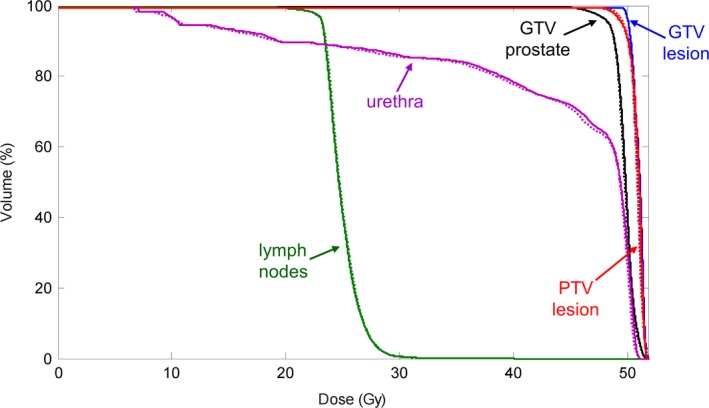
Dose volume histograms of the dose distributions calculated for the prostate VMAT patient case. Solid lines: calculated in a commercial TPS (Varian Eclipse); Dotted lines: dose calculated in our goMC dose engine with the commissioned beam model.

The doses calculated for a VMAT prostate patient case treated with 10 MV FFF beam are shown in Fig. 9. The first two rows represent the dose calculated in the Eclipse TPS and the one calculated in our goMC dose engine, respectively. The absolute differences between these two doses are shown in the third row. The dose volume histograms corresponding to these two doses are shown in Fig. 10. Both figures show a good agreement between the Eclipse‐calculated dose and our calculated dose. A 3D gamma‐index test was performed to quantitatively evaluate the discrepancy between the two doses. The passing rate was 99.82% for 2%/2 mm criterion and 95.71% for 1%/1 mm criterion. These results have demonstrated that with the developed commissioning and source sampling approaches, our current goMC dose engine achieves clinically acceptable accuracy for treatment plan dose calculation.

We would like to mention that we used a prostate case instead of a HN case when comparing our goMC with the Eclipse TPS. The reason for not using a HN case was because Eclipse used the Analytical Anisotropic Algorithm (AAA) for dose calculation, which was known to be inaccurate in complex tissue heterogeneity. Dose differences between MC and AAA have been reported for HN cases in other studies.^27^, ^28^

## 4 Discussion and conclusions

We have presented our recent improvements on a GPU‐OpenCL‐based MC dose engine goMC for its use in the clinic. First, several modifications were made on our automatic beam commissioning approach that was previously developed for a phase‐space‐let beam model, not only to adapt this approach to the new analytical beam model used in goMC, but also to streamline the commissioning process for clinical use. Second, we also performed comprehensive tests on six real linac photon beams used in our institution, including different beam energies and FFF beams, which have demonstrated the general validity and feasibility of our auto‐commissioning approach and the analytical beam model for clinical use. Third, we utilized FMs to bias the control point sampling for source particles already sampled from our beam model. This method could reduce the portion of the particles that would hit MLC leaves and contribute little to patient dose, leading to more effective use of sampled source particles in computational intensive MC particle transport. This effect led to smaller statistical uncertainty level, improving the overall efficiency of the dose calculations for VMAT treatment plans. Our FM‐based biased sampling method is applicable to both IMRT and VMAT treatment plans, although it is particularly beneficial for VMAT cases. We also compared our calculated dose with the Eclipse‐calculated dose for a VMAT prostate patient case. The good agreement between the two doses has demonstrated that the overall accuracy achieved by our GPU‐based goMC dose engine is acceptable for clinical use.

It actually took us two steps to reach an accurate analytical beam model to represent an actual linac beam: (1) fitting the particle distribution of a phase‐space file into an analytical beam model first; (2) further fine tuning the beam model by adjusting the relative intensities of the sub‐sources to match the measured data. It would be preferred to fit the analytical model directly using the measured dose. However, the relationship between the particle distribution and the resulting dose is complicated. It is very difficult to directly model this relationship in a closed analytical form and then use it to fit the beam model. Our current two‐step strategy is an alternative way to overcome these challenges. It is relatively straightforward to analyze the particle distribution of a phase‐space file, choose proper analytical functions, and then fit the model parameters to faithfully replicate this distribution. This step is expected to give us a beam model close enough to a real clinical beam. Then starting with a distribution already close to the true solution, we only need to finely tune the energy spectrum and fluence distribution of our beam model through commissioning using the dose measured in water. This fine tuning is actually a linear problem, and is expected to result in a reliable and physically reasonable beam model. Because our MC dose engine models the physical interactions of the radiation beam in different materials, once the energy spectrum and fluence distribution of the beam model is finely tuned to match the dose measured in water, the commissioned model would hold for heterogeneous medium.

We assumed beam rotational symmetry when deriving our analytical beam model from a phase‐space file and further commissioning it. The reason was twofold. One was to efficiently use the limited amount of the particles stored in the file to build a model with relatively small statistical uncertainty. Another reason was to decrease the number of correction factors to be adjusted during commissioning. However, the actual spot size of the photon beam was usually found to be elliptical.^21^ This elliptical photon spot, as well as different locations of X jaws and Y jaws along beam direction, should result in the non‐interchangeable output factors of rectangular shapes^29^ and the different penumbras of the inline and cross‐line dose profiles. Our goMC dose engine considered the different locations of X and Y jaws, and achieved a good match with the measured inline and cross‐line profiles. In addition, for rectangular fields, both the output factors calculated by our commissioned model and the measured output factors are non‐interchangeable, and they agree well with each other. For example, for two rectangular open fields, e.g., 6 × 15 cm^2^ and 15 × 6 cm^2^, the output factors of the commissioned 6 MV FF beam model were 0.992 and 0.984, compared to the measured values of 0.996 and 0.987. These results indicate that the beam asymmetric issue above the jaw does not seem to be a major concern for clinical use, although further studies are needed to test this in more challenging cases, e.g., small field dosimetry, with more stringent criteria.

Although we aimed to realize the clinical use of goMC, the auto‐commissioning method and the biased particle sampling strategy for treatment plan dose calculation are not specific to our own dose engine. First, our idea to solve the beam commissioning problem from an optimization perspective provides a general auto‐commissioning approach. Our commissioning idea may be applicable to other linac beams, such as Cyberknife. Second, the proposed FM‐based biased source sampling method is expected to be applicable to a variety of beam models. For instance, a phase‐space file beam model presents the same efficiency issue for VMAT dose calculation, and may benefit from our sampling method.

## Conflict of interest

The authors have no relevant conflicts of interest to disclose.
